# Defect Induced Polarization Loss in Multi‐Shelled Spinel Hollow Spheres for Electromagnetic Wave Absorption Application

**DOI:** 10.1002/advs.202004640

**Published:** 2021-02-08

**Authors:** Ming Qin, Limin Zhang, Xiaoru Zhao, Hongjing Wu

**Affiliations:** ^1^ MOE Key Laboratory of Material Physics and Chemistry under Extraordinary School of Physical Science and Technology Northwestern Polytechnical University Xi'an 710072 China

**Keywords:** Co‐based spinel MCo_2_O_4_, defect engineering, electromagnetic wave absorption, multi‐shelled hollow spheres, polarization loss

## Abstract

Defect engineering is an effective approach to manipulate electromagnetic (EM) parameters and enhance absorption ability, but defect induced dielectric loss dominant mechanism has not been completely clarified. Here the defect induced dielectric loss dominant mechanism in virtue of multi‐shelled spinel hollow sphere for the first time is demonstrated. The unique but identical morphology design as well as suitable composition modulation for serial spinels can exclude the disturbance of EM wave dissipation from dipolar/interfacial polarization and conduction loss. In temperature‐regulated defect in NiCo_2_O_4_ serial materials, two kinds of defects, defect in spinel structure and oxygen vacancy are detected. Defect in spinel structure played more profound role on determining materials’ EM wave dissipation than that of oxygen vacancy. When evaluated serial Co‐based materials as absorbers, defect induced polarization loss is responsible for the superior absorption performance of NiCo_2_O_4_‐based material due to its more defect sites in spinel structure. It is discovered that electron spin resonance test may be adopted as a novel approach to directly probe EM wave absorption capacities of materials. This work not only provides a strategy to prepare lightweight, efficient EM wave absorber but also illustrates the importance of defect engineering on regulation of materials’ dielectric loss capacity.

## Graphical abstract

The importance of defect induced polarization loss in electromagnetic wave absorption is revealed by engineering defect in serial Co‐based multi‐shelled spinel hollow spheres. For the first time, defect in spinel structure and its induced loss mechanism is found to play dominate role over other loss mechanisms. This unique hollow structure also affords lightweight spinel electromagnetic wave absorbing materials.

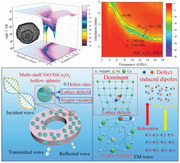

## 1 Introduction

In recent years, development of efficient electromagnetic (EM) wave absorbing materials to eliminate excessive EM wave interfere has caused great research attention.^[^ ^1^, ^2^, ^3^, ^4^, ^5^, ^6^, ^7^, ^8^, ^9^ ^]^ Co‐based spinel structured metal oxides are regarded as prospective candidates as effective EM wave absorbing materials. To date, a series of MCo_2_O_4_ (M = Fe, Ni, Mn, etc.) based EM wave absorbers have been prepared and exhibit promising absorption behaviors.^[^ ^10^, ^11^, ^12^, ^13^, ^14^, ^15^, ^16^, ^17^, ^18^ ^]^ Though these advances are inspiring, the high density issue of MCo_2_O_4_ based EM wave absorbers still hinders their practical applications. More importantly, researches on these MCo_2_O_4_ absorbers are individual and a systematic research on these serial EM wave absorbing materials to reveal their connections and differences on EM wave attenuation has not been reported yet. Based on above discussion, it is highly desired to design a specific morphology to reduce the high density issue of MCo_2_O_4_‐based EM wave absorbers. At the meantime, a systematic study on series of MCo_2_O_4_ EM wave absorbers to comprehensively disclose their attenuation behaviors and mechanisms is of great significance.

Development of hollow structured materials has been regard as an attractive solution to solve high density issue of materials.^[^ ^19^, ^20^, ^21^ ^]^ As an attractive micro/nanostructure, multi‐shelled hollow spheres (MSHS) have aroused extensive attention due to its unique features such as lightweight, controllable shell numbers and thickness, shell porosity, and intershell spacing.^[^ ^22^, ^23^, ^24^, ^25^, ^26^, ^27^ ^]^ It can be foreseen that this peculiar structure is favorable for promoting the EM wave absorption performance by following aspects: 1) the multiple shells would lead to the multiple reflections when incident EM wave propagates in the cavities between two adjacent shells; 2) the abundant interfaces generated on the shell–air–shell could boost the interfacial polarization; 3) due to the existence of multiple cavities in this structure, its low density and lightweight features can precisely circumvent density issue of MCo_2_O_4_‐based EM wave absorbers. These merits of MSHS‐based materials are expected to enable the generation of high‐performance EM wave absorbing materials with low density. However, researches on MSHS‐based EM wave absorbers are still insufficient. Therefore, more efforts should be devoted on this research aspect.

Apart from the morphology regulation, another issue that we confront is the quantitative relationship and preferred dissipation mechanism in dielectric loss materials since the coexistence of multiple loss mechanisms (e.g., interfacial polarization, dipolar polarization, defect polarization, and conduction loss). Previous researches have devoted efforts to design peculiar structure to distinguish the dominant loss mechanism.^[^ ^28^, ^29^, ^30^, ^31^, ^32^ ^]^ For example, Ji's group reported that interfacial polarization loss dominant graphene/ferrite composite EM wave absorbers and tuning its intensity by manipulating the contact area between graphene and ferrite. By contrast, investigation on defect engineering and its induced polarization loss dominant EM wave absorbers is paid less attention. Let alone currently reported defects engineering investigation such as oxygen vacancy and defect in carbon materials could not be quantitative due to the uncertain contribution from other mechanisms. Thus, it is imperative attempt to clarify the EM wave absorbing mechanism dominantly caused by defect induced polarization loss.

Inspired by this thought, we reported a versatile route for synthesis of multi‐shelled MCo_2_O_4_ based hollow spheres structured materials. Through identical synthetic route, samples with same triple‐shelled structure were readily attained, which was significant for reducing the materials’ density and eliminating the influence of other loss mechanisms on EM wave dissipation. With respect to spinel structure MCo_2_O_4_, the variance of bivalent metal ions can lead to diverse defect degree due to their different properties, which is suitable for investigation of defect polarization. We first selected NiCo_2_O_4_‐based materials as representative and manipulated their defect in crystal and oxygen vacancy through calcination temperature regulation. When evaluated serial NiCo_2_O_4_‐based materials as EM wave absorbers, their suitable morphology and phase composition design helped us exclude other EM wave absorption mechanisms. Through in‐depth analyses of materials defect and oxygen vacancy, we clarified that the defect in spinel structure of NiCo_2_O_4_‐based absorbers induce polarization loss determined absorbers’ dissipation capacity for the first time. By extending to serial Co‐based MSHS materials under optimal defect site engineering temperature, their EM wave absorption performance still followed the order of defect site in spinel structure, verifying the importance of defect engineering on regulating spinel ferrites’ EM wave consumption ability. In addition, though not determining factor, polarization loss induced by oxygen vacancy also played positive role on enhancement of Co‐based spinel absorbers’ capacity. In summary, the ingenious design of MHSH structure not only solves the high density issue of ferrite but also sheds light on the defect induced polarization loss dominant mechanism in spinel ferrite for the first time.

## 2 Results and Discussion

**Scheme 1:**
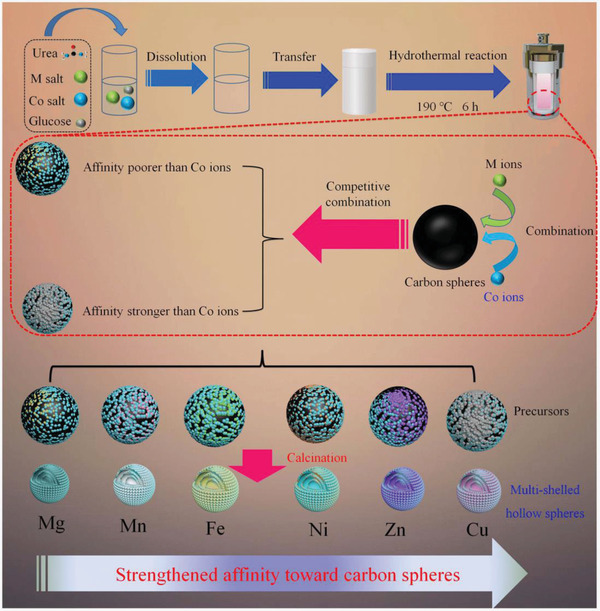
Schematic of the synthetic process for the multi‐shelled hollow spheres.

The synthetic processes for multi‐shelled MCo_2_O_4_ hollow spheres are depicted in **Scheme** **1**. The metal sources, glucose, and urea were jointly dissolved into the deionized water and the resultant homogeneous solution was directly used for the hydrothermal reaction without further treatment (e.g., pH adjustion) to prepare precursors. Finally, calcination in air would remove the carbon templates in the samples and lead to the formation of porous multi‐shelled MCo_2_O_4_ hollow spheres. Even though the products with analogous multiple layers structure can be observed by changing the bivalent metal ions, they exhibit different elements and crystalline phases. Thus, a competitive combination mechanism was proposed and shown in the synthetic schematic (Scheme 1) and will be discussed in next paragraph.

**Figure 1:**
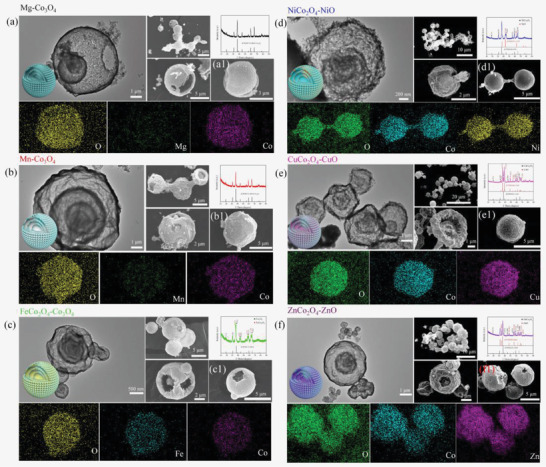
SEM, EDS mapping, XRD patterns and TEM images of products obtained by a) Mg, b) Mn, c) Fe, d) Ni, e) Cu, and f) Zn ions as reactants, the EDS mappings correspond to a1–f1) of the SEM images.

We tried six kinds of bivalent metal ions, that is, Mg, Mn, Fe, Ni, Cu, and Zn to construct multi‐shelled MCo_2_O_4_ by the identical synthetic route. The morphologies of these samples were present in **Figure** **1**a–f, order of which followed with above metal ions species. We can learn that all the prepared samples display well‐defined spherical and multiple layers structure according to scanning electron microscope (SEM) and transmission electron microscope (TEM) images. However, X‐ray diffraction (XRD) patterns and energy dispersive spectroscope (EDS) mapping images of samples demonstrate that the elements distribution and crystalline phases are quite different from each other (Table S1, Supporting Information). Based on the results, we suspect that competitive combination between carbon spheres and metal ions occurred during the hydrothermal process. When Mg and Mn is added into the reaction system, the carbon spheres prefer to combine with Co ions and only small amount of Mg and Mn ions enters into carbon spheres. Thus the XRD patterns of calcined products displayed identical diffraction peaks compared to Co_3_O_4_ with minor red shift due to the smaller ionic radius of Mg and Mn, which could be regarded as doping. When Fe, Ni, Cu, and Zn were added, though the addition of M and Co was stoichiometric ratio according to MCo_2_O_4_ and the uniformly spatial distribution of these elements were confirmed, all the XRD patterns of above samples were multiphase. For Fe^2+^ ions addition, it contained FeCo_2_O_4_ and Co_3_O_4_ with comparable diffraction peaks intensity, suggesting the combination between carbon spheres and Co^2+^ ions is stronger than that of Fe^2+^. While for Ni^2+^ ions addition, NiO phase appears but the main phase is NiCo_2_O_4_. With respect to Zn^2+^, the ZnCo_2_O_4_ and ZnO phases are comparable. And it turned out that the CuO became the main phase when Cu^2+^ was adopted into reaction system. Thus, we suspect that competitive combination between M and Co^2+^ ions occurred during reaction and the intensity of affinity followed the order of Mg^2+^ < Mn^2+^ < Fe^2+^ < Co^2+^ < Ni^2+^ < Zn^2+^ < Cu^2+^. Based on above analysis, we propose a feasible formation mechanism for these Co‐based materials, which can be described as follow: under the hydrothermal reaction, the d‐glucose in the solution gradually forms carbon spheres due to the dehydration and carbonization. The original carbon spheres seeds tend to bond with the metal ions through the electrostatic force between the negatively charged carbon and positively charged ions. Simultaneously, abundant functional groups including —OH and —COO— also contribute to the combination of carbon matrix and metal ions. However, due to the different properties of metal ions, competitive combination mechanism between carbon spheres and metal ions occurred. The carbon spheres would combine preferentially with specific metal ions, leading to the non‐stoichiometric loading amount of metal ions. As the reaction prolonging, the primary carbon spheres would be encapsulated by the newly formed carbon spheres, resulting in the formation of core–shell structure. After several repeating processes, the multi‐shell precursors are attained. The final heat treatment is utilized to eliminate the carbon and convert the non‐crystalline metal moiety into desired Co‐based binary spinel materials. Due to the varied contents in carbon spheres, the calcined samples exhibited diverse crystalline phases instead of standard single phased corresponding MCo_2_O_4_.

To best of our knowledge, currently reported researches on general route for synthesis of serial MSHS ferrite are rare.^[^ ^33^, ^34^, ^35^, ^36^, ^37^ ^]^ Moreover, their methods are either tedious (over 1 day) or expensive on carbon source choice. These drawbacks would hinder their applications. In comparison, the synthetic period of this route can be significantly reduced, which can cut down the consumption of energy and promote productivity. In addition, cheaper d‐glucose is chosen as carbon source, beneficial to decline the cost of experiment. Above merits bring about a low‐cost and eco‐friendly method for the fabrication of serial MSHS ferrite materials.

**Figure 2:**
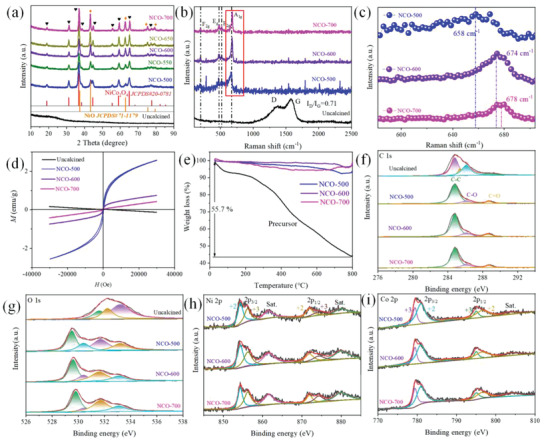
a) XRD patterns of as‐prepared samples, b) Raman spectra, c) magnified image in red square of (b), d) magnetization curves, and e) thermogravimetry curves of uncalcined sample, NCO‐500, NCO‐600, and NCO‐700, and their corresponding high‐resolution XPS spectra for f) C 1s, g) O 1s, h) Ni 2p, i) Co 2p.

The NiCo_2_O_4_ based materials calcined at different temperatures were selected as the representative to investigate the influence of defect induced polarization loss on materials’ EM wave absorption capacity. XRD patterns of these samples are present in **Figure** **2**a. For the uncalcined one, the pattern displays the obviously amorphous carbon peak, which is broad and centers at 20°. No other distinguish peak can be found in the pattern, which may be ascribed to the fact the other components are wrapped by the carbonaceous spheres. After heat treatment at different temperatures, the typical diffraction peaks of spinel structure NiCo_2_O_4_ can be seen for all the samples. As calcination temperatures increased, the diffraction peaks also become sharper, revealing the better crystalline NiCo_2_O_4_. In addition, NiO phase is also detected in these NiCo_2_O_4_ samples obtained at different calcination temperatures. This may be ascribed to the fact that the different combining capacity between the carbon template and metal ions.^[^ ^38^ ^]^

To further investigate the structure information of as‐prepared samples, Raman spectra were carried out and shown in Figure 2b,c. Likewise, the uncalcined sample displays different characteristic peaks compared with others. The distinguishable peaks at 1378 and 1575 cm^−1^ are assigned to the D band and G band of carbon, which stand for the degree of defect and order in carbonaceous materials, respectively. The ratio between intensity of D band and G band is 0.71, demonstrating the low graphitization degree of the uncalcined sample. This result is in good agreement with the amorphous carbon in XRD pattern. When being subjected to heat treatment, a clear phase transformation can be observed. The Raman spectra of calcined samples exhibit typical characteristic peaks of spinel materials. Namely, the peaks locate at 189, 475, 520, and ≈674 cm^−1^ are ascribed to the phonon modes of spinel NiCo_2_O_4_. It also should be noted that as the temperature gradually increased, the peak that represents symmetric Co^3+^‐O bond in octahedral exhibits a blue shift toward higher wave number from NCO‐500 (NiCo_2_O_4_ precusor calcined at 500 °C) of 658 cm^−1^ to NCO‐700 of 678 cm^−1^, which can be clearly observed in Figure 2c. When crystal defects in spinel structure break the symmetry in the lattice, location of this peak would shift to lower wave number.^[^ ^39^, ^40^, ^41^ ^]^ In view of their values, the crystal defects in as‐prepared samples decreased from NCO‐500 to NCO‐700. This may be attributed to that higher calcination temperature leads to higher crystalline and less defects in spinel structure. However, the characteristic peak of NiO is absent in the Raman spectra, which may be ascribed to the low signal intensity of NiO compared to baseline. Moreover, characteristic peaks of carbon are extinct in calcined samples, implying complete removal of carbon spheres template.

Elements and their chemical states were further investigated through X‐ray photoelectron spectroscopy (XPS) and shown in Figure 2f–i. The XPS surveys confirm the presence of C, O, Co, and Ni elements in the calcined samples while only C and O are detected in the uncalcined one. After the deconvolution of Co 2p and Ni 2p spectra of the samples, these metal elements are found to be existed in multiple valence states of +2 and +3 (see Figure 2h,i). This result can be well fitted with previous researches about elements analysis. The detail information about the ratio of different valence states of metal ions is calculated based on their peak area. One can see that both of Co^2+^ and Ni^2+^ ions gradually decrease while Co^3+^ and Ni^3+^ ions increase with the elevated calcination temperatures (Table S2, Supporting Information). This is due to that the metal ions are oxidized in air at high temperature. Based on the previous researches, the decline in Co^2+^/Co^3+^ ratio in spinel structured materials indicates the decrease of oxygen vacancies.^[^ ^13^ ^]^ However, the percentage of oxygen vacancies obtained from XPS result in Figure 2g is not in accordance with the Co^2+^/Co^3+^ ratio. Among the investigated samples, NCO‐700 possesses the highest oxygen vacancies concentration. It has been reported that high temperature is beneficial to the formation of oxygen vacancies in NiO species.^[^ ^42^, ^43^ ^]^ Thus, the variance of oxygen vacancies in NiO/NiCo_2_O_4_ composite is collectively determined by NiO and NiCo_2_O_4_. Noteworthy, both of the polarization loss induced by defects in spinel structure and oxygen vacancy could contribute to dielectric loss in NCO EM wave absorbing materials. Due to their dissimilar variation tendency, we can confirm the dominant factor by investigating their EM wave absorption performance. Overall, the highly consistent characterization results of XRD, XPS, and Raman spectra collectively confirm the successful synthesis of NiO/NiCo_2_O_4_ samples.

The field dependence of magnetization for NCO specimens measured at room temperature was recorded in Figure 2d. As can be seen, the uncalcined sample exhibits diamagnetic behavior. Abovementioned results indicate that only non‐magnetic carbon component exists in sample. Thus, it is understandable that the non‐magnetic substance would display diamagnetic behavior.^[^ ^44^ ^]^ After heating treatment, specimens possess ferromagnetic curves due to ferromagnetic nature of spinel NiCo_2_O_4_ that is formed after calcination. Though the magnetization shows a decrease tendency with elevating calcination temperature, the maximum magnetization of NCO‐500 is only 3.78 emu g^−1^. The rather low magnetization of NCO samples is unable to provide strong magnetic loss. Thus, we can infer the EM wave dissipation of NCO is mainly caused by dielectric loss, which can also be verified by loss tangent value latter.

Thermogravimetric curves of representative samples are present in Figure 2e. For the precursors, weight loss appeared throughout the whole heating process. The first weight loss region from start to 200 °C is caused by the removal of adsorbed water in the samples. Typically, the carbon spheres can be totally removed between 400 to 500 °C according to previous researches.^[^ ^45^, ^46^, ^47^ ^]^ However, a successive mass loss can be observed along with the increasing temperature beyond 500 °C, which may be attributed to the decomposition of NiCo_2_O_4_ on higher temperature. Through this weight loss curve, we could learn that dipoles such as adsorbed water molecule and polar functional group in precursors can be removed at 500 °C. Thus, the dipole polarization in the calcined samples is inconspicuous and the temperature of 500 °C can be adopted for the preparation of NCO samples. With regard to the NiO/NiCo_2_O_4_ samples harvested at different temperatures, they only show slight weight loss from room temperature to 800 °C. Due to the dielectric loss‐dominant feature, NCO EM wave absorbers can also work under high temperature without concerning lose their magnetic properties over the Curie temperature. Combined with its high heat stability, NCO MSHS displays great potential for high‐temperature EM wave absorption applications.

**Figure 3:**
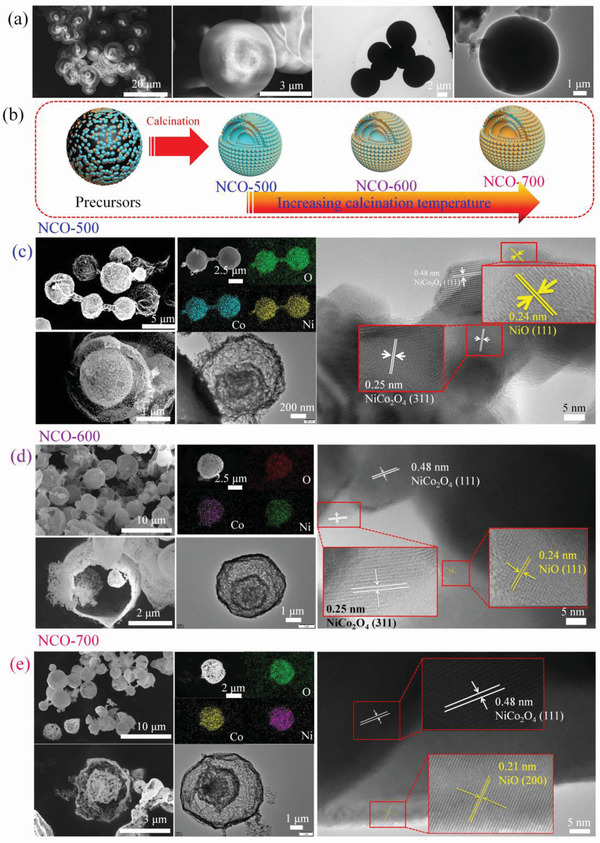
SEM, EDS mapping, TEM and HR‐TEM images of a) uncalcined sample, c) NCO‐500, d) NCO‐600, e) NCO‐700, and b) its synthetic mechanism.

The morphologies of these samples were viewed by field‐emission scanning electron microscope (FE‐SEM). For the precursors, it displays solid spherical structure with smooth surface (**Figure** **3**a). The spheres possess uniform size with diameter around 4.7 µm. The uniform geometry and size distribution make it a valid template for the synthesis of multi‐shelled Co‐based spinel hollow spheres. After heating treatment, the solid carbon spheres transform into multi‐shelled hollow spheres structure (see Figure 3b). Though the products retain the spherical shape, abundant pores, and folds are in the presence of the shell. The pores may arise from the release of CO_2_ gas which results from carbon spheres combusting under heating treatment. The magnified FESEM images clearly reveal that the outmost shell of these spheres is constructed by considerable loosely packed nanoparticles. The broken spheres in Figure 3c confirms the NCO‐500 possess a thin shell and a core. The core has a diameter of ≈1.55 µm. As a whole, the diameter of complete spheres of NCO is 2.5 µm, much smaller than the pristine carbon spheres (as schematic in Figure 3b). This phenomenon is also caused by the removal of carbon spheres during the calcination, resulting in the shrinkage of residual substance. NCO spheres with multilayer structure can also be observed through the porous outmost and inner shells and corresponding broken spheres with elevated calcination temperatures in Figure 3d,e. It should also be pointed out that fragments and irregular spheres are in the presence of high‐temperature treated samples, demonstrating partial collapse of the shell architectures. The Ni, Co, and O elements in the calcined samples can be clearly visualized without any distinguishable elemental segregation according to EDS mapping, demonstrating the formation of homogeneous oxides. Except for the representative samples, the NCO harvested at 550 and 650 °C also showed same complex inner structure and well distributed elements (Figures S1 and S2, Supporting Information).

To have a deeper insight into the inner structure of as‐obtained samples, their morphologies are further inspected by TEM and shown in Figure 3 (uncalcined sample and samples calcined at 500, 600, and 700 °C are selected as examples). For the uncalcined one, colloidal carbon spheres could be found in its TEM images. Diameter of colloidal carbon sphere is measured to be ≈5 µm, well fitted with the results from SEM images. After heating treatment, samples with multi‐shelled hollow spheres structure can be readily observed. A maximum 3 layers of shell is present in the TEM images of calcined samples. Abundant inner cavities and thin thickness of inner shells (≈100 nm) ensure this material with ultralight feature, which is beneficial to significantly reduce the density of corresponding EM wave absorbers. Moreover, distinctive lattice fringes can be observed on the high‐resolution TEM images. Taking NCO‐500 as example, the interplanar spacing are measured to be 0.48, 0.25, and 0.24 nm, which can be well indexed to the (111) and (311) crystal planes of NiCo_2_O_4_ and (111) crystal planes of NiO, respectively. The coexistence of NiCo_2_O_4_ and NiO phases can also be detected in other two samples at higher calcination temperature. To sum up, the variance on calcination temperature leads to identical triple‐shelled hollow spheres and components (i.e., NiCo_2_O_4_ and NiO) but different component ratios (as schematic in Figure 3b). As mentioned earlier, interfacial polarization is primarily caused by the aggregation of polarized charges at the interfaces with different conductivities or polarities. On the serial NCO composites, though the intrinsic conductivities or polarities of NiCo_2_O_4_ and NiO are invariable, their ratio changed due to the diverse calcination temperatures. In addition, NiCo_2_O_4_ and NiO are uniformly distributed on the whole sphere, thus the interfaces between NiCo_2_O_4_ and NiO would increase due to the emerging and augment of NiO phase. As a result, dielectric loss contributed from interfacial polarization of heterojunction interfaces between NiCo_2_O_4_ and NiO would strengthen owing to the enlarged contact area. Despite the varied contribution from interfacial polarization loss, the influence of morphology on EM wave absorption can be excluded due to their identical triple‐shelled hollow sphere structure.

According to above discussion, we discover that the calcination temperature plays more important role on determining the configuration of final products. The contents of different components, defects in spinel structure and oxygen vacancies can be modulated by different temperatures. As a contrast, its influence on shell numbers, space of adjacent shells, and shell porosity is not profound. In consequence, the influence of morphology on EM wave consumption behaviors may not be distinguishable due to the quite approximate shell parameters. For a dielectric loss dominated EM wave absorbing materials, attenuation of EM wave by serial NCO samples would come from interfacial polarization, dipolar polarization, conduction loss, and defect induced polarization loss. However, the high calcination temperature of NCO samples can eliminate the inherent dipoles including water molecule and functional groups, thus dipole polarization in these samples can be ruled out. In consequence, other three mechanisms are the potential causes for EM wave absorption. Interestingly, we discovered that the interfacial polarization loss intensity induced by the NiCo_2_O_4_ and NiO phases is strengthened due to the their extended contact area at ascending calcination and conduction loss delivered the same trend owing to the promoted conductivities (described later). In contrast, defects and its induced polarization loss displayed exactly the reverse tendency in serial NCO absorbing materials. This phenomenon provides the chance to validate the prominent loss mechanism of defect induced polarization in NCO samples by comparing their variance trend of EM wave absorption performance in next two paragraphs. Despite its insufficient influence on EM wave absorption, multi‐shelled structure's lightweight feature is highly needed in EM wave absorption application.

The EM parameters are present in Figure S5, Supporting Information. Since NCO samples are dielectric loss dominated EM wave absorbers, more attention is focused on complex permittivity of serial NCO samples (depicted in Figure S5a, Supporting Information, b). The real part and imaginary part *ε*′ and *ε*″ values stand for the dielectric energy storage and attention capacity. One can learn that *ε*′ and *ε*″ values of precursors are around 2.8 and 0.2, respectively. The low complex permittivity is ascribed to the absence of crystalline NiCo_2_O_4_, implying the poor dielectric loss capacity. When the calcination temperature is 500 °C, the sample possessed the highest *ε*′ and *ε*″ values against other samples. The corresponding values range from 11.0 to 3.7 and 2.8 to 6.4, respectively. It should be noted that three distinctive resonance peaks located at 14.7, 15.9, and 17.0 GHz can be observed in the frequency versus *ε*″ curve. These resonance peaks may be related to the multiple polarization relaxation process originated from defect induced polarization in NCO samples, which is further confirmed by Cole–Cole semicircles in Figure S6, Supporting Information. As the calcination temperature gradually increased, the *ε*′ and *ε*″ values of samples are found to decline, suggesting poor EM wave dissipation capacities. This result proves that interfacial polarization cannot be the predominant loss mechanism. Otherwise, NCO650 and NCO700 are supposed to possess higher *ε*″ values than that of others. Though the resonance peaks are also appeared on other samples, descending *ε*″ values indicate the weakened EM wave absorption capacities. This result can be further illuminated by dielectric loss tangent as shown in Figure S5c, Supporting Information.

The EM wave dissipation capacities of NiCo_2_O_4_‐based absorbers were studied. Detailed reflection loss (RL) values are calculated and drawn in Figures S3 and S4, Supporting Information. For the uncalcined material, it displayed the poorest EM wave absorption performance. The absence of NiCo_2_O_4_ crystalline phase and low conductivity originated from low graphitization degree of carbonaceous component in precursor together led to poor EM dissipation capacity. With respect to calcined samples, we could find that the EM wave consumption capacity of the as‐obtained samples gradually decreased along with increasing temperature. As mentioned earlier, EM wave attenuation from interfacial polarization loss and dipole polarization loss can be excluded in serial NCO samples. Except for above loss mechanisms, conduction loss may also be the leading source for EM wave attenuation. To uncover the contribution from conduction loss, the electron transport characteristics of serial NCO samples were measured and shown in Figure S7, Supporting Information. In Nyquist plots, diameter of semicircle represents electronic transfer resistance and the smaller diameter indicates the higher conductivity.^[^ ^30^ ^]^ On basis of their curves, the semicircle shows downward trend along with increasing calcination temperature, implying the conductivity gradually increase from NCO500 to NCO700. However, higher conductivity of NCO650 and NCO700 cannot bring high EM wave absorption capacities for these samples, implying conduction loss is not the dominated loss mechanism. Based on above discussion, we confirm that defect and its induced polarization loss is the exclusive cause connected to EM wave absorption capacity. However, there are two kinds of defects in NiO/NiCo_2_O_4_ composite, namely, crystal defect and oxygen vacancy. According to the variance tendency, if the oxygen vacancy induced polarization is dominant factor, the EM wave performance would first decrease from NCO‐500 to NCO‐600 and then further increase for NCO700 according to XPS analysis. Unfortunately, NCO‐700 exhibited the worst performance, suggesting oxygen vacancy cannot be the dominant factor. Conversely, EM wave absorption performance behaved exactly the same trend as the variance tendency of defect in spinel structure, that is, both of the EM wave absorption performance and crystal defect gradually decrease from NCO‐500 to NCO‐700. Therefore, it demonstrates that defect in spinel structure and its induced polarization loss is the dominant mechanism that can determine the absorption capacity of NCO absorbers. After the validation of this defect induced polarization loss, another idea comes to our mind, may this model be adopted on other multi‐shelled Co‐based hollow sphere structured materials?

**Figure 4:**
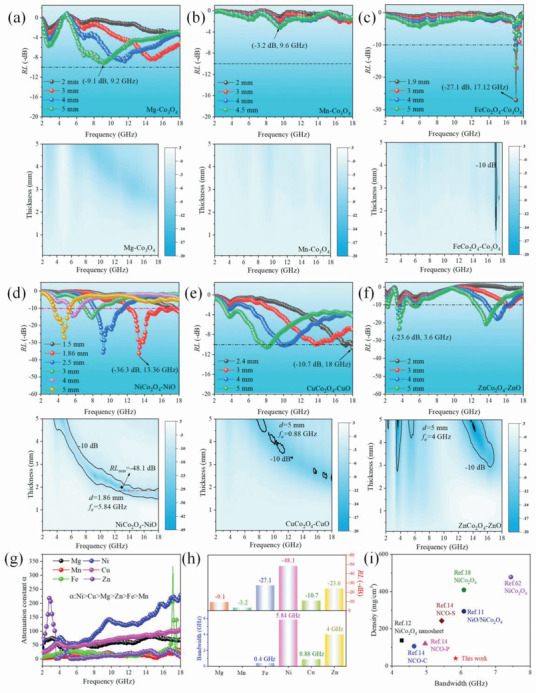
a–e) RL value verse frequency and corresponding 2D plots with different thickness for samples obtained from different divalent metal ions a) Mg^2+^, b) Mn^2+^, c) Fe^2+^, d) Ni^2+^, e) Cu^2+^, and f) Zn^2+^, g) attenuation constants, h) summary of optimal EM wave RL values and corresponding *f*_E_ values, i) density versus bandwidth characteristics of most NiCo_2_O_4_ samples.

To validate whether this mechanism is suitable in other ferrites, the EM wave absorption performance of other Co‐based spinel multi‐shell hollow spheres prepared under optimal condition (highest defects site at 500 °C) was also investigated. The detailed information can be seen in **Figure** **4**. For the single‐phased Co_3_O_4_, RL values cannot reach to −10 dB on whole 2–18 GHz, which can be visualized from Figure 4a,b. Even though, RL values of Co_3_O_4_ obtained from Mg is slightly higher than that of obtained from Mn. With regard to the multi‐phase samples, EM wave absorption capacity is promoted and the minimum RL (RL_min_) value can reach to the desired one of −10 dB. From Figure 4d, the RL_min_ of CuCo_2_O_4_/CuO increases to −10.7 dB while the effective bandwidth (*f*_E_) is narrow and less than 1 GHz. FeCo_2_O_4_/Co_3_O_4_ displays the similar behavior but its RL_min_ is further improved to −27.1 dB, much higher than the former one. In contrast, its *f*_E_ is extremely narrow in high frequency, as can be seen in Figure 4c. As for ZnCo_2_O_4_/ZnO in Figure 4f, the EM wave attenuation capacity is dramatically improved. RL_min_ of −23.6 dB can be acquired with thickness of 5 mm and *f*_E_ is as wide as 4 GHz, located at four separated locations. Among them, NiO/NiCo_2_O_4_ exhibits the best EM wave absorption in all aspects. Wide *f*_E_ of 5.84 GHz from 12.16 GHz to 18 GHz can be fulfilled at thin thickness of 1.86 mm. At the same time, a strong absorption peak of −48.1 dB is achieved at thickness of 1.9 mm. Detailed information about RL_min_ values and effective bandwidth is given in Figure 4h. It is arrestive that great property discrepancy occurred among these structure‐ and morphology‐analogical EM wave absorbers, which has not been systematically reported before. Through the regular attenuation constant (Figure 4g) and impedance match characteristic of samples (Figures S8–S11, Supporting Information), it is indubitable that NiO/NiCo_2_O_4_ would display the best EM wave absorption capacity due to its highest attenuation constant value and well matched impedance. However, deeper sight into the cause of NiO/NiCo_2_O_4_’s excellent EM wave dissipation capacity should also be figured out.

**Figure 5:**
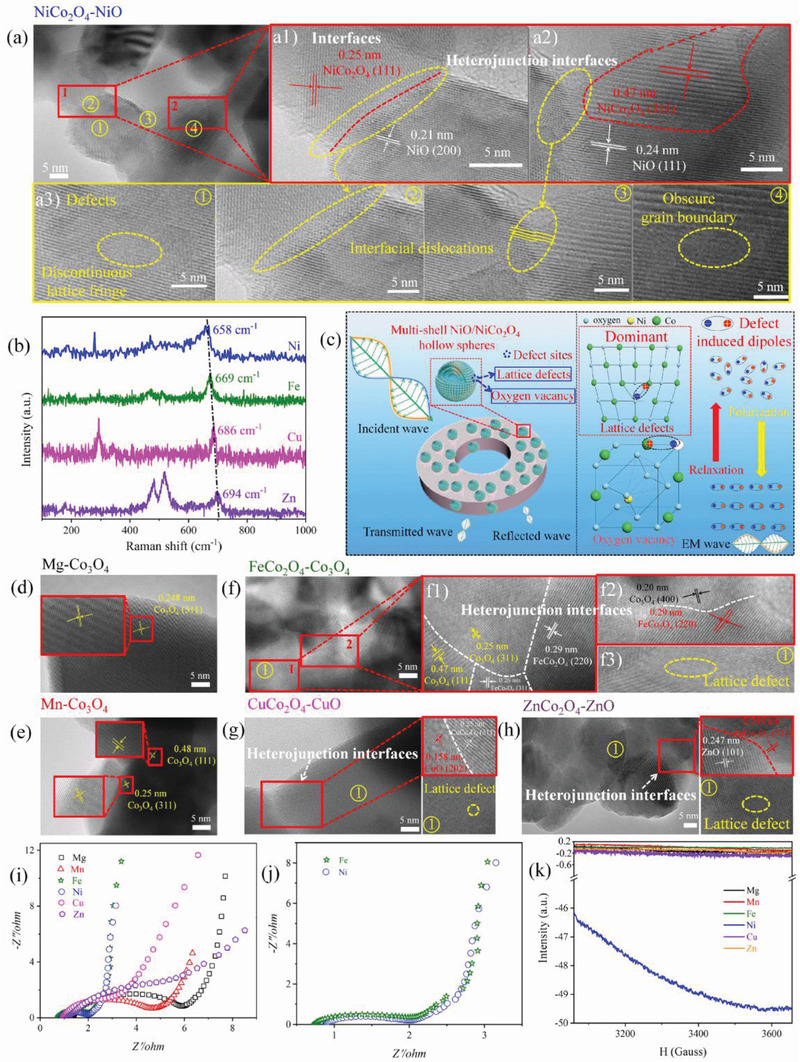
HR‐TEM and corresponding interfaces and defects in MCo_2_O_4_ composites. a–a3) NiCo_2_O_4_‐NiO, b) Raman spectra of Fe, Ni, Cu, and Zn ions as reactants, c) EM wave absorption mechanism, d) Mg‐Co_3_O_4_, e) Mn‐Co_3_O_4_, f–f3) FeCo_2_O_4_‐Co_3_O_4_, g) CuCo_2_O_4_‐CuO, h) ZnCo_2_O_4_‐ZnO, i,j) EIS, and k) ESR curves of serial Co‐based spinel samples.

To elucidate the superb EM wave absorption behaviors of serial Co‐based spinel samples, we have carefully investigated the influence of products’ structure, defects, oxygen vacancies on its dielectric loss capacity. Based on XRD patterns, the samples obtained with Mg and Mn are single‐phased Co_3_O_4_. Compared with other multiple phased products, the interfaces significantly declined, which can be inspected by high‐resolution TEM images in **Figure** **5**. TEM images of single‐phased Co_3_O_4_ harvested by Mg and Mn show a pure single crystal without evidence of interfaces and defect sites (Figure 5d,e). Obviously, the absence of multiple interfaces and defects in these samples cannot induce interfacial polarization and defect polarization, leading to poor dielectric loss capacity and inferior EM wave absorption behaviors. Magnetic loss is also weak in these two samples in view of their poor magnetic loss tangent (Figure S11b, Supporting Information). Thus, their EM wave attenuation capacities are originated from conduction loss or defect induced polarization by oxygen vacancies. Therefore, we have checked the XPS spectrum of O 1s for Co_3_O_4_ (Figure S12, Supporting Information). The peak located around 531.6 eV assigned to oxygen vacancy in specimens prepared by Mg is higher than that of Mn, implying stronger dipolar polarization can be induced in Mg‐doped samples. While for conduction loss, the conductivity of Mn‐doped samples is higher than Mg‐doped samples according to EIS measurement (Figure 5i), suggesting higher conduction loss can be provided by Mn‐doped samples. On basis of their EM wave absorption performance, contribution from oxygen vacancy induced polarization loss instead of conduction loss is more profound so that the absorption capacity followed the variance trend of oxygen vacancy in these single‐phased Co_3_O_4_ samples. The results that conduction loss is not the prominent loss mechanism can be well fitted with the conclusion of serial NCO samples. Thus, we can infer that dipolar polarization induced by oxygen vacancy is dominant dielectric loss in Co_3_O_4_ with poor magnetic loss, interfacial polarization and defect polarization.

With respect to other four multi‐phase samples, the interfaces including homogeneous interface and heterojunction interfaces between MCo_2_O_4_ and MO are remarkably increased due to the contacts of different phases. Distinctive interfaces between different phases and lattice planes are visualized and marked by red square in corresponding high‐resolution transmission electron microscopy (HR‐TEM) images in Figure 5a1,a2,f1,f2,g,h. Therefore, the existence of heterojunction interfaces between different crystalline phases in these composites can give rise to interfacial polarization. According to Che's research, the accumulation of polarized charges is caused by the different work functions of each component.^[^ ^31^, ^48^, ^49^ ^]^ To reach a Fermi level equilibrium, the polarized charge electrons would flow to component with higher work function. Thus we collect work function of corresponding metal oxides ^[^ ^50^, ^51^, ^52^, ^53^ ^]^ and discover their differences are not profound (Table S3, Supporting Information). Therefore, the accumulated charge density may not be dramatically changed on the heterojunction interfaces of different phases. Moreover, the geometry of samples is the same as triple‐shelled hollow sphere structure. These factors lead to negligible difference in polarization loss intensity of multiphase samples.

Similar to serial NCO samples, EIS of serial Co‐based ferrite materials were also measured and depicted in Figure 5i,j. We can see that different metals will dramatically change the conductivity of samples. Among these materials, FeCo_2_O_4_ and NiCo_2_O_4_ samples show much smaller diameters than others and their data even overlap in many data points on the magnified curves (Figure 5j), implying the conductivities of FeCo_2_O_4_ and NiCo_2_O_4_ are comparable. However, significantly different EM wave absorption performances were obtained from these samples. This result confirms that conduction loss is not the dominated loss mechanism in serial Co‐based ferrite EM wave absorbing materials.

To illuminate the connection between crystal defect and EM wave absorption capacity of the multi‐phased samples, we have carefully checked their HR‐TEM images to find out their defect sites and marked by yellow circles with numbers in Figure 5a3,f3,g,h. The discontinuous lattice fringes are caused by edge dislocations and the stacking lattice is caused by interfacial dislocations in order to accommodate the difference in lattice parameter at the coherent interface.^[^ ^54^, ^55^, ^56^, ^57^, ^58^, ^59^ ^]^ Among these materials, NiO/NiCo_2_O_4_ composite has more obscure lattice fringes, implying the existence of abundant defects on the surface of samples. In addition, lattice mismatch between different phases of NiO and NiCo_2_O_4_ also gives rise to abundant defects around their transition stages. The detail information can be observed in Figure 5a3. These all contribute to massive defect sites and undoubtedly are conductive to induce strong polarization loss. These defects would break the lattice period and tune electric field and electric transport path. Thus, the existed defects would hinder the movement of free electrons and give rise to aggregate charges. The defect in spinel crystal can also be evident by Raman spectra (Figure 5b). As mentioned earlier, peak located at ≈660 cm^−1^ is assigned to symmetric Co^3+^‐O bond in octahedral site of spinel structure and shift of its location implies the degree of lattice defects. Clearly, the location of this peak follows the order of Ni < Fe < Cu < Zn, and red shift indicated the idea of the more crystal defects. All these proofs confirm NCO samples have the highest defect sites in spinel structure. As a result, NCO sample showed the strongest EM wave dissipation capacity, which is determined by its highest polarization loss induced by plentiful defects.

Though oxygen vacancy induced polarization loss is not the main contribution for the exceptional EM wave absorption capacity of absorbers, it is undoubtedly high‐concentration oxygen vacancies is beneficial to result in higher polarization (as evident in single‐phased Co_3_O_4_ samples). In general, the oxygen vacancy on the samples can act as dipoles to generate dipole polarization. On basis of high‐resolution O 1s spectra (Figure S13, Supporting Information), deconvolution result reveals that NCO possesses the high oxygen vacancy concentration. Therefore, local defect dipoles can be induced under external EM field. These dipoles are disordered but would arrange orderly along the propagation direction of the applied EM field. The transition between ordered and disordered dipoles also effectively dissipates EM wave energy. Thus, the high oxygen vacancy concentration in NCO samples also brings about the strong polarization loss. By combining above analyses, we confirm that defect in spinel structure and its induced polarization loss are still the leading factor on determining EM wave absorbers’ dissipation capacity on serial Co‐based ferrite. With regard to another type of defect, oxygen vacancy is also positively related to the superior EM wave absorption performance of NiO/NiCo_2_O_4_ composite. To sum up, both of the polarization loss induced by defects in spinel structure and oxygen vacancy, namely, defect induced polarization loss mechanism result in the outstanding EM wave absorption performance of multi‐shelled NiO/NiCo_2_O_4_ hollow sphere composite against others.

Electron spin resonance (ESR) test was conducted to uncover the oxygen vacancies in serial Co‐based ferrite samples. In general, resonate peaks in ESR curve can be discerned due to the electrons trapped on oxygen vacancies.^[^ ^60^, ^61^, ^62^ ^]^ However, the peaks were absence in curves but an interesting phenomenon was discovered instead. It should be noted that ESR is not only known to be a very sensitive method to probe paramagnetic centers but also to microwave absorption.^[^ ^63^ ^]^ For a high dielectric loss material, it will distort the microwave field inside an ESR resonator, which results in distortion of the ESR signal from spins inside the material.^[^ ^64^ ^]^ The samples to be tested in this work have the potential to dissipate the energy of EM wave by dielectric loss, which may influence the test signals. In our test results, the data in test curves are basically negative due to their consumption of EM wave energy, leading to the lack of peak values (Figure 5k). Moreover, the negative values are proportional to their EM wave attenuation capacities. Among serial Co‐based samples, NCO500 clearly displayed the lowest values and the curve even changed into arch. This result suggests the strongest EM wave absorption capacity of NCO500, which is in line with our conclusion in this work. In short, ESR technique may be not suitable to investigate the oxygen vacancy in EM wave absorbers but can be a useful tool to validate their absorption abilities. This finding may also provide us a more visualized and facile approach to pick out materials with strong EM wave absorption capacities rapidly.

Through the serial characterization results, solid evidences have been found to explain the outstanding EM wave absorption performance of NiO/NiCo_2_O_4_ composite. The detail reasons could be concluded as follows.

Defect induced polarization loss, including defect in crystal structure and oxygen vacancy, is the main loss mechanism that leads to the excellent EM wave absorption capacity of multi‐shelled Co‐based spinel hollow spheres. Thereinto, defect in spinel structure and its induced polarization loss play more profound role on determining EM wave absorption capacity. Among serial Co‐based EM wave absorbers, more defects are visualized from HR‐TEM images and evidently demonstrated by Raman spectra in NiO/NiCo_2_O_4_ composite. Defects on samples can trap free electrons and break the balance of spacial charge distribution. Under external EM field, dipole moments are generated and subsequent relaxation process can effectively dissipate energy carried by EM wave (Figure 5c). Thus, more defects can offer NiO/NiCo_2_O_4_ composite strengthened defect induced polarization loss than that of others (Figures S6 and S14, Supporting Information), resulting in the strongest EM wave absorption performance.

Besides, oxygen vacancy induced polarization loss can also contribute to the superior EM wave dissipation of NiO/NiCo_2_O_4_ composite. In virtue of XPS technique, oxygen vacancy on surface of NiO/NiCo_2_O_4_ composite is measured to be 32.3%. High‐concentration oxygen vacancies can act as electric dipoles under EM field. The ordered and disordered transition of electric dipoles also brings about EM wave energy consumption.

To have a better view of our absorbers’ EM wave absorption behaviors, we have collected other NiCo_2_O_4_‐based and multi‐shelled hollow spheres structured EM wave absorbing materials.^[^ ^65^, ^66^, ^67^ ^]^ The comparison is listed in **Table** **1**. Apparently, our multi‐shelled NiCo_2_O_4_/NiO hollow spheres absorber displays wide effective absorption bandwidth of 5.84 GHz with thin coating thickness of 1.86 mm.

**Table 1:** The comparison of previous researches on multi‐shelled hollow sphere‐based and NiCo_2_O_4_‐based EM wave absorbers with our work

Absorbers	Optimal RL_min_ [dB]	*f*_E_ and the corresponding thickness [GHz] (RL ≤ −10 dB)	Refs.
Multi‐shelled conductive polymer	−39.7	NA	^[^ ^65^ ^]^
Multi‐shelled Ni/NiO	−7	–	^[^ ^66^ ^]^
C@NiCo_2_O_4_@Fe_3_O_4_	−43.0	2.10 (3.4 mm)	^[^ ^15^ ^]^
NiCo_2_O_4_/CoNiO_2_	−42.13	3.92 (1.55 mm)	^[^ ^13^ ^]^
NiCo_2_O_4_ nanosheet	−42.9	4.28 (1.39 mm)	^[^ ^12^ ^]^
NiCo_2_O_4_/Co_3_O_4_/NiO	−28.6	4.76 (1.6 mm)	^[^ ^16^ ^]^
NiCo_2_O_4_	−39.4	7.44 (2.1 mm)	^[^ ^67^ ^]^
MnO_2_@NiCo_2_O_4_	−58.4	2.7 (4 mm)	^[^ ^17^ ^]^
NiO/NiCo_2_O_4_ microrod	−57.4	6.08 (1.88 mm)	^[^ ^18^ ^]^
Multi‐shelled NiO/NiCo_2_O_4_ hollow spheres	−48.1	5.84 (1.86 mm)	This work
	−37.5	5.92 (2.14 mm)	

Though the high EM wave absorption performance of NiO/NiCo_2_O_4_ composite is induced by defects induced polarization loss, interfaces polarization and conduction loss, its unique multi‐shelled hollow spheres morphology should also be emphasized. As mentioned, multi‐shelled hollow spheres structure endows structure lightweight feature. Thus, we calculate the density of multi‐shelled NiO/NiCo_2_O_4_ hollow spheres and make a comparison with our previously prepared NiCo_2_O_4_‐based EM wave absorbing materials (depicted in Figure 4i and listed in Table S4, Supporting Information). The density of current material is ≈41.1 mg cm^−3^, which is the lowest against others. In view of the low density, wide EM wave absorption bandwidth in *Ku* band (5.84 GHz from 12.16 to 18 GHz) at relatively thin thickness (1.86 mm), the lightweight and efficient multi‐shelled NiO/NiCo_2_O_4_ hollow spheres EM wave absorber may be used in practical application field of satellite broadcast devices and aviation communication devices.

## 3 Conclusion

In summary, a low‐cost and eco‐friendly approach for the general preparation of serial multi‐shelled Co‐based MCo_2_O_4_ hollow spheres was conducted. It was found that different bivalent metal ions showed varied affinity toward carbon spheres template compared with Co ions, leading to formation of multi‐shelled hollow spheres with diverse crystalline phase. By in‐depth investigation of the composition, conductivity, and defect sites in ferrites, EM wave dissipation from conduction loss, interfacial polarization, and dipolar polarization were found to play lesser important role than defect induced polarization loss, which is beneficial to investigate the influence of defect engineering on dielectric loss dominant mechanism absorbers. By selecting NiO/NiCo_2_O_4_ composite as representative, we found between two kinds of defects, namely, crystal defect in spinel structure and oxygen vacancy, contribution from the former one is more profound thus EM wave dissipation behaviors followed its variance tendency. To validate this discovery, we also investigated the EM wave absorption performance of serial Co‐based MSHS materials. Through in‐depth analyses of characterization results, we still found that NiO/NiCo_2_O_4_ composite possessed the highest defect sites in spinel structure, thus behaving the best EM wave absorption performance. In virtue of unique multi‐shelled hollow spheres structure, we demonstrated the defect induced dielectric loss dominant mechanism for the first time. Still, we discovered that signal of ESR measurement is proportional to EM wave absorption capacities of materials to be measured, which may be developed as a novel approach to rapidly pick out excellent EM wave absorbers. The finding may offer inspirations to fabricate high‐performance and lightweight ferrite EM wave absorbers by defect engineering.

## 4 Experimental Section

##### Reagents

Metal salts including nickel nitrate hexahydrate (Ni(NO_3_)_2_·6H_2_O), cobalt nitrate hexahydrate (Co(NO_3_)_2_·6H_2_O), copper nitrate trihydrate (Cu(NO_3_)_2_·3H_2_O), zinc nitrate hexahydrate (Zn(NO_3_)_2_·6H_2_O), manganese acetate tetrahydrate (Mn(CH_3_COO)_2_·4H_2_O), magnesium sulfate heptahydrate (MgSO_4_·7H_2_O), ferrous chloride tetrachloride (FeCl_2_·4H_2_O), d‐glucose, and urea were purchased from National Reagent Corp. (Shanghai, China). The above chemicals were directly used in experiments without further purification.

##### Synthesis of Multi‐Shelled NiO/NiCo_2_O_4_ Hollow Spheres

In a typical synthetic process, 2.5 mmol of Ni(NO_3_)_2_·6H_2_O, 5 mmol of Co(NO_3_)_2_·6H_2_O, 6 g d‐glucose and 7.5 mmol of urea were together added into a beaker. Thereafter, 60 mL of deionized water was poured into above baker and the mixture was converted into homogeneous solution by 30 min of magnetic stirring. Then the solution was transferred into 100 mL of Teflon‐lined autoclave and kept 190 °C for 6 h. Precursors were collected by lavation using deionized water and ethyl alcohol alternately and placed into drying oven for 12 h. To gain final products with different defects and oxygen vacancies, precursors were calcined in muffle furnace with varied temperatures of 500, 550, 600, 650, and 700 °C while the heating rate was identical 2 °C min^−1^. The samples attained at different temperatures were labeled as NCO‐500, NCO‐600, NCO‐550, NCO‐650, and NCO‐700, respectively.

##### Synthesis of Other Co‐Based Multi‐Shelled Hollow Spheres

For the synthesis of other Co‐based multi‐shelled hollow spheres, the synthetic route and the dosage were identical to NiCo_2_O_4_ except for that the Ni metal salt was replaced by other metal salts (e.g., Mg, Mn, Fe, Cu, and Zn).

##### Characterization

The crystalline structures of as‐prepared samples were tested by XRD. More detail structure information was recorded by Raman spectra. The chemical composition and element valance states were measured by XPS. Magnetic properties were examined by physical property measurement system at room temperature. Morphologies of samples were inspected by FE‐SEM and HR‐TEM. The bulk density of materials was obtained from specific value of powder's mass to the certain bulk it filled. The electrochemical impedance spectroscopy of samples was conducted in a typical three‐electrode cell containing a working electrode, a platinum (Pt) plate (3 cm × 4 cm) counter electrode, and a Hg/HgO (3 m KOH, aqueous) reference electrode. In this test, working electrode was prepared by spreading samples on Ni foam (1 cm × 1 cm) by mixing with PVDF and acetylene black with mass ratio of 8:1:1. EIS test was in frequency range of 10^−1^–10^5^ Hz. The ESR was performed at 77 K on a Bruker EC500 X‐band spectrometer. To evaluate the EM wave absorption capacity of samples, 50 mg of as‐prepared samples and 50 mg of paraffin wax were homogeneously mixed and pressed into mold to prepare coaxial ring with inner and outer diameters of 3.04 and 7.00 mm, respectively. The EM parameters were measured by vector network analyzer in frequency range of 2–18 GHz. The corresponding RL values were calculated based on transmission line theory, which was expressed in following formulas:

(1) $$\mathrm{RL}=20\log_{10}\left|\frac{Z_{\mathrm{in}}-Z_{0}}{Z_{\mathrm{in}}+Z_{0}}\right|$$

(2) $$Z_{\mathrm{in}}=\sqrt{\frac{\mu_{r}}{\varepsilon_{r}}}\tan\;h\left(j\frac{2\pi fd}{c}\sqrt{\mu_{r}\varepsilon_{r}}\right)$$

In these equations, *Z*_in_ and *Z*_0_ are the input impedance of absorbing materials and the impedance of free space, *d* is the thickness of the absorbing materials, *c* is the speed of light in vacuum, *f* is the frequency, *ε*_r_ and *μ*_r_ are the complex permittivity and permeability, respectively.

## Conflict of Interest

The authors declare no conflict of interest.

## Supporting information

Supporting Information
