# Supplementary material for: Defect Induced Polarization Loss in Multi‐Shelled Spinel Hollow Spheres for Electromagnetic Wave Absorption Application
Source: Adv Sci (Weinh). 2021 Feb 8;8(8):2004640. doi: 10.1002/advs.202004640 (PMC8061380; doi:10.1002/advs.202004640)
Supplement: Supplementary file 1 — Supporting Information [file ADVS-8-2004640-s001.pdf]

© 2020 Wiley-VCH GmbH

ADVANCED SCIENCE Open Access

Supporting Information

for *Adv. Sci.*, DOI: 10.1002/advs.202004640

Defect Induced Polarization Loss in Multi-shelled Spinel Hollow Spheres for Electromagnetic Wave Absorption Application

*Ming Qin, Limin Zhang, Xiaoru Zhao, Hongjing Wu**

Supplementary information:

Defect induced polarization loss in multi-shelled spinel hollow spheres for electromagnetic wave absorption application

Ming Qin, Limin Zhang, Xiaoru Zhao, Hongjing Wu*

MOE Key Laboratory of Material Physics and Chemistry under Extraordinary, School of Physical Science and Technology, Northwestern Polytechnical University, Xi'an 710072, China;

* Corresponding author: wuhongjing@nwpu.edu.cn (H. Wu).

Table S1 The products prepared by different bivalent metal ions

Bivalent metal ions	Mg	Mn	Fe	Ni	Cu	Zn
Products	Co_3O_4	Co_3O_4	$FeCo_2O_4$<br>Co_3O_4	NiO<br>$NiCo_2O_4$	CuO<br>$CuCo_2O_4$	ZnO<br>$ZnCo_2O_4$

Table S2 The calculated results of the Co^{2+} ratios and oxygen vacancies form XPS peak areas

Samples	Co^{2+}/%	Co^{3+}/%	Co^{2+}/ Co^{3+}	Ni^{2+}/%	Ni^{3+}/%	Oxygen vacancy/%
NCO-500	72.9	27.1	2.70	59.3	40.7	32.3
NCO-600	72.6	27.4	2.66	54.0	46.0	31.5
NCO-700	64.9	35.1	1.85	47.7	52.3	35.9

Table S3 Work function of materials and their differences collected from reference

Materials	Work function (eV)	Work function difference (eV)	Refs.
$NiCo_2O_4$	6.1	-	[50]
NiO	5.4	-	
$NiO/NiCo_2O_4$	-	0.7	
$CuCo_2O_4$	4.87	-	[52]
CuO	5.31	-	[53]
$CuO/CuCo_2O_4$	-	0.44	
ZnO	4.45	-	[51]
$ZnCo_2O_4$	5.22	-	
$ZnO/ZnCo_2O_4$	-	0.77	

Table S4 Comparison with our previously prepared $NiCo_2O_4$-based EM wave absorbing materials

EM wave absorbers	Bandwidth (GHz)	Thickness (mm)	Density (mg/cm^{-3})	Refs.
$NiCo_2O_4$ nanosheet	4.28	1.39	137	[12]
NCO-S	5.44	1.8	243	
NCO-P	4.64	1.6	106	[14]
NCO-C	4.96	1.9	120	
$NiCo_2O_4$	6.08	2.06	409	[18]
NiO/$NiCo_2O_4$ microrod	6.08	1.88	294	[11]
$NiCo_2O_4$	7.44	2.1	478	[62]
**Multi-shelled NiO/$NiCo_2O_4$ hollow spheres**	**5.84**	**1.86**	**41.1**	**This work**

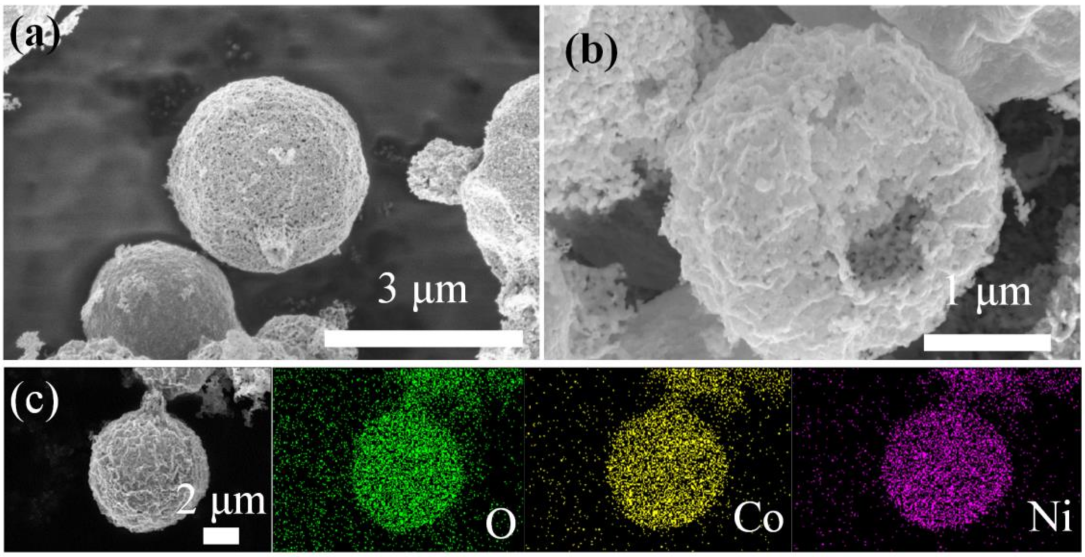

Fig. S1 SEM images and EDS mapping of NCO-550.

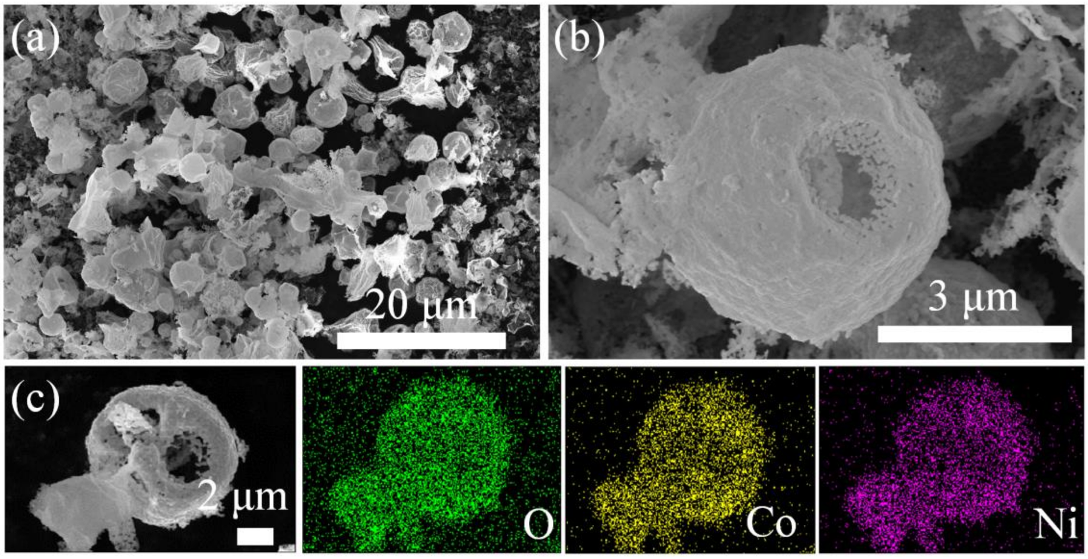

Fig. S2 SEM images and EDS mapping of NCO-650.

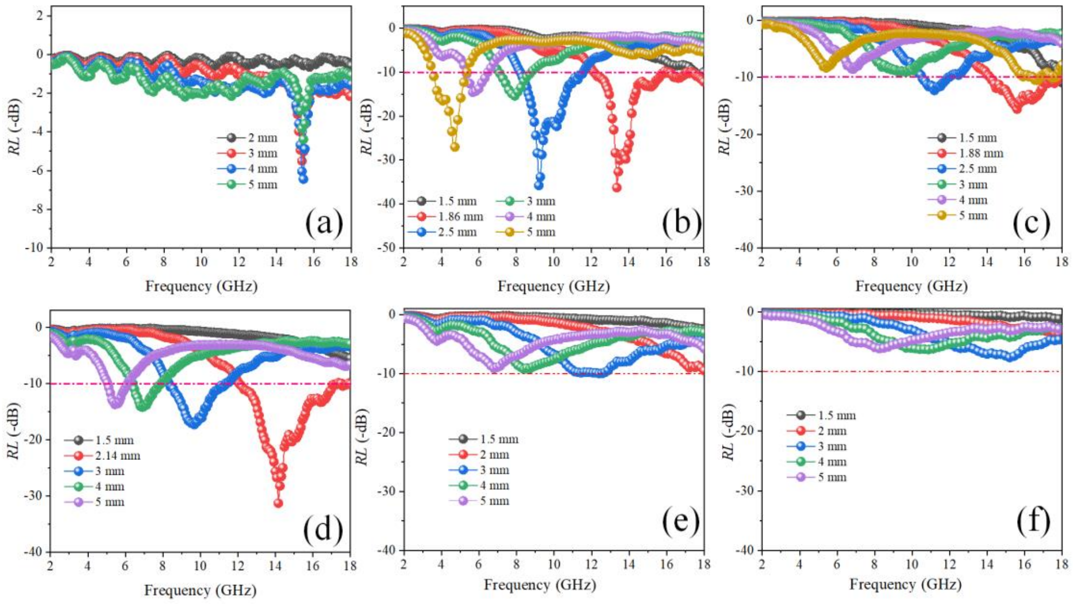

Fig. S3 *RL* values of (a) $NiCo_2O_4$ precursors, (b) NCO-500, (c) NCO-550, (d) NCO-600, (e) NCO-650 and (f) NCO-700 as a function of frequency.

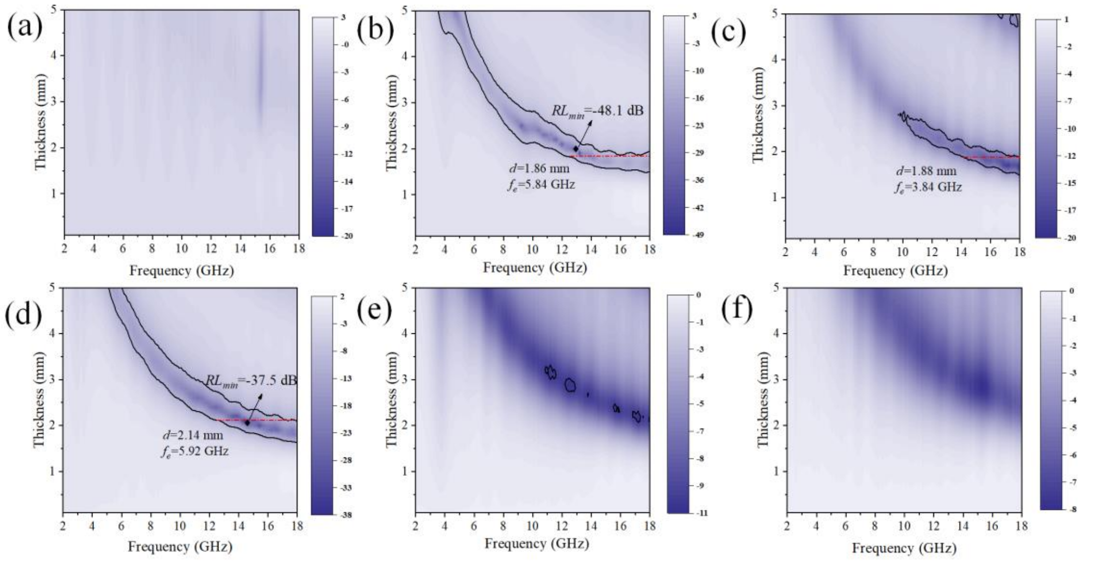

Fig. S4 2D plot of (a) $NiCo_2O_4$ precursors, (b) NCO-500, (c) NCO-550, (d) NCO-600, (e) NCO-650 and (f) NCO-700 as a function of frequency.

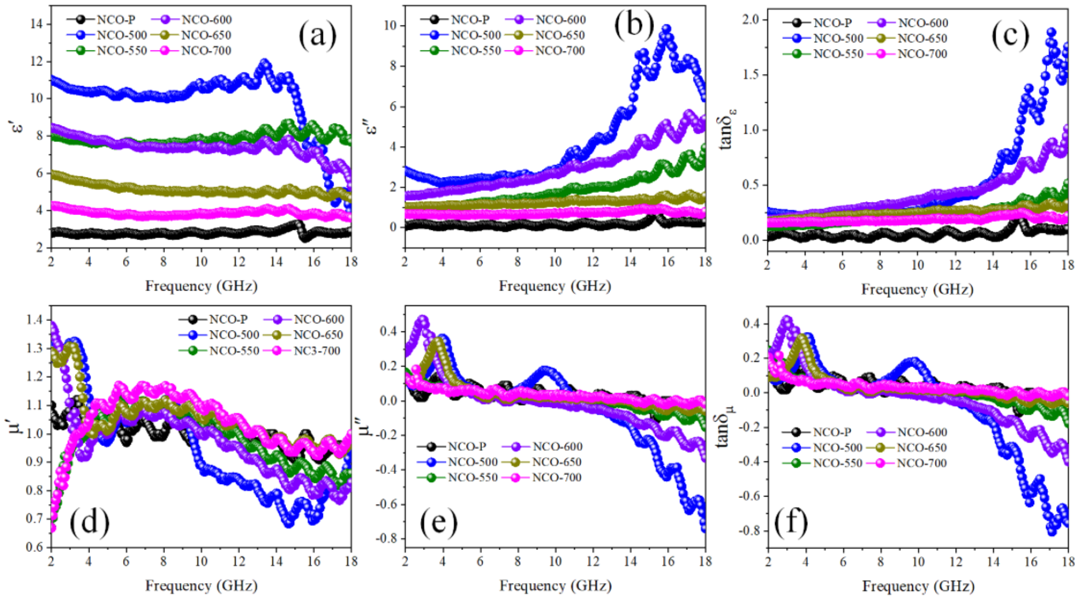

Fig. S5 (a) The real part and (b) imaginary part of complex permittivity, (c) the dielectric loss tangent, the (d) real part, (e) imaginary part of complex permeability, (f) the magnetic loss tangent of the as-obtained NCO samples.

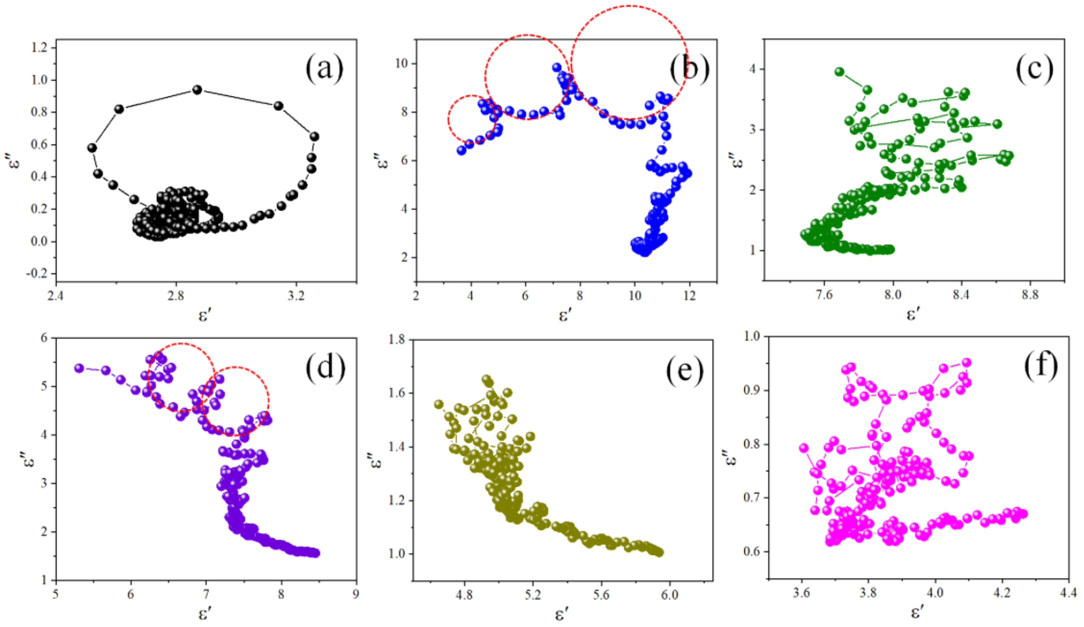

Fig. S6 *Cole-Cole* semicircles of (a) $NiCo_2O_4$ precursors, (b) NCO-500, (c) NCO-550, (d) NCO-600, (e) NCO-650 and (f) NCO-700.

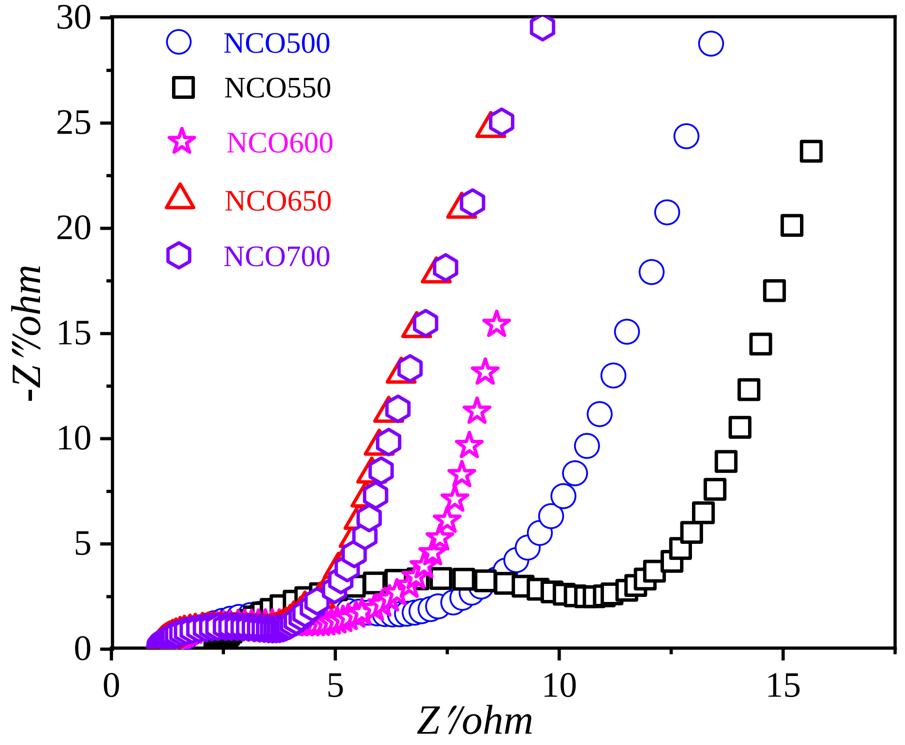

Fig. S7 EIS curves of serial NCO samples.

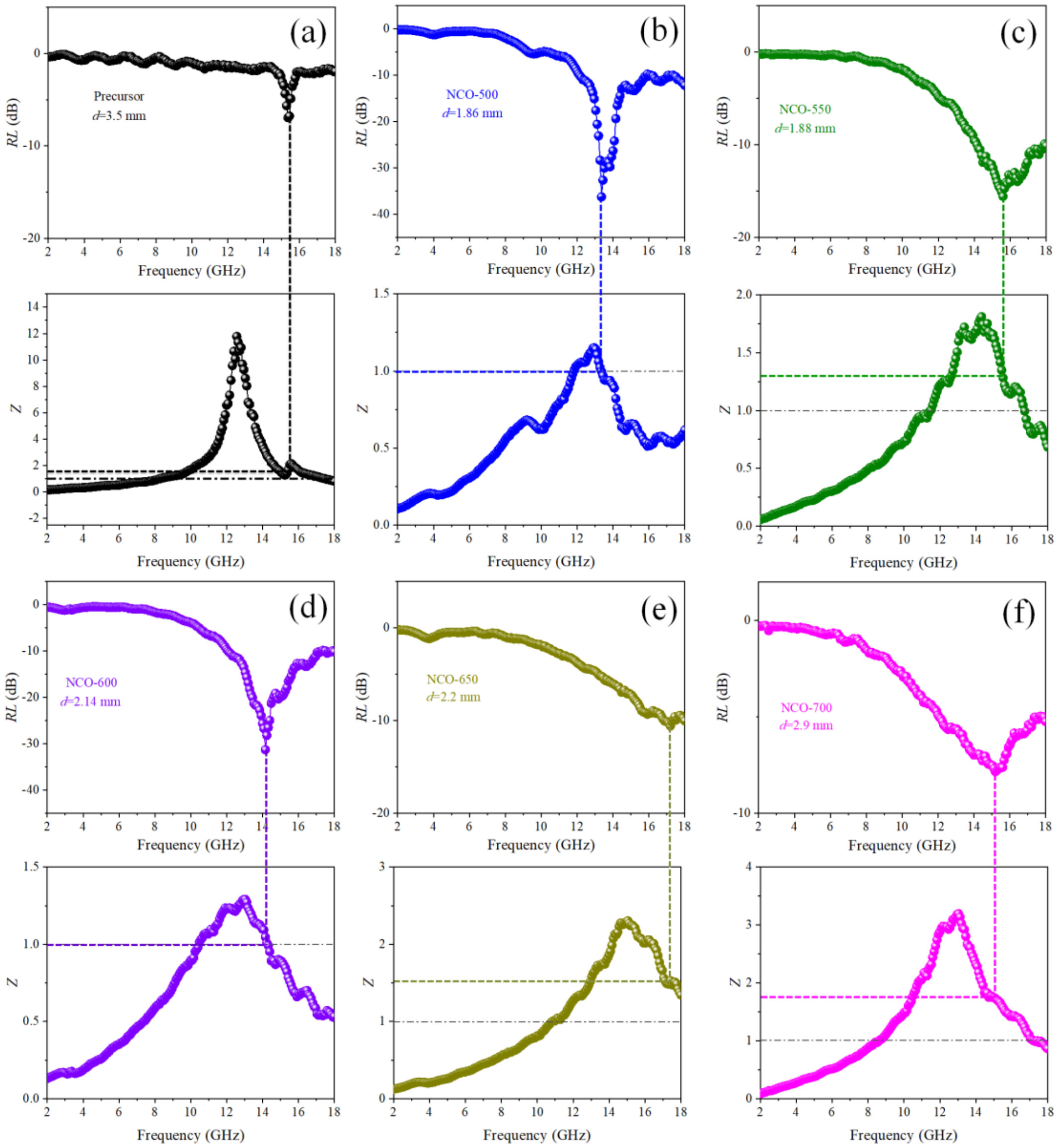

Fig. S8 Normalized impedance matching characteristic of NCO samples.

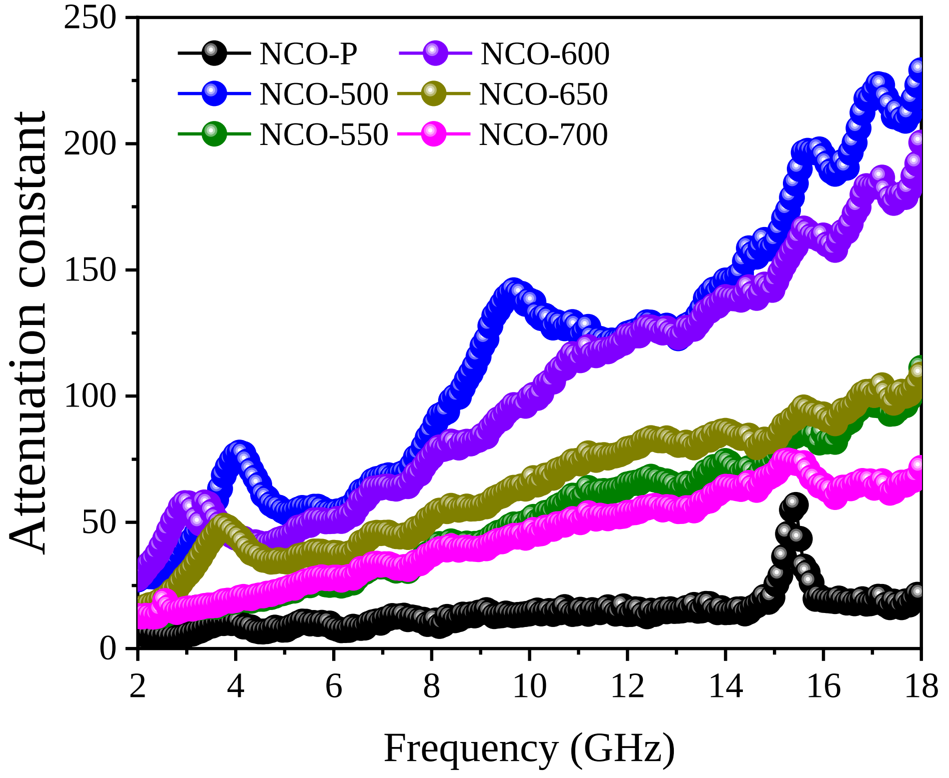

Fig. S9 Attenuation constant of as-prepared NCO samples.

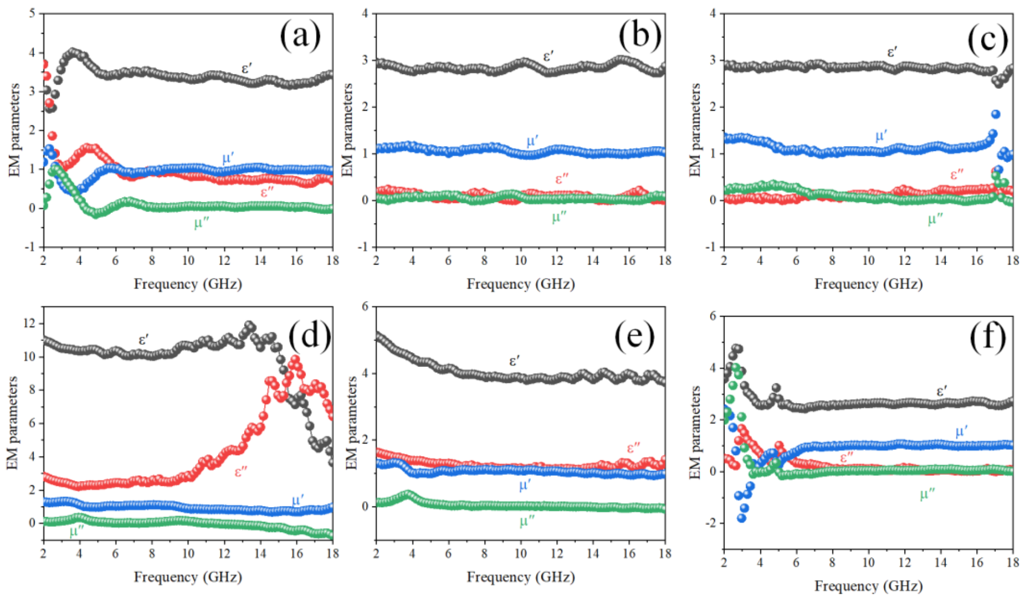

Fig. S10 EM parameters of samples obtained from different divalent metal ions (a) Mg^{2+},(b) Mn^{2+}, (c) Fe^{2+}, (d) Ni^{2+}, (e) Cu^{2+} and (f) Zn^{2+}.

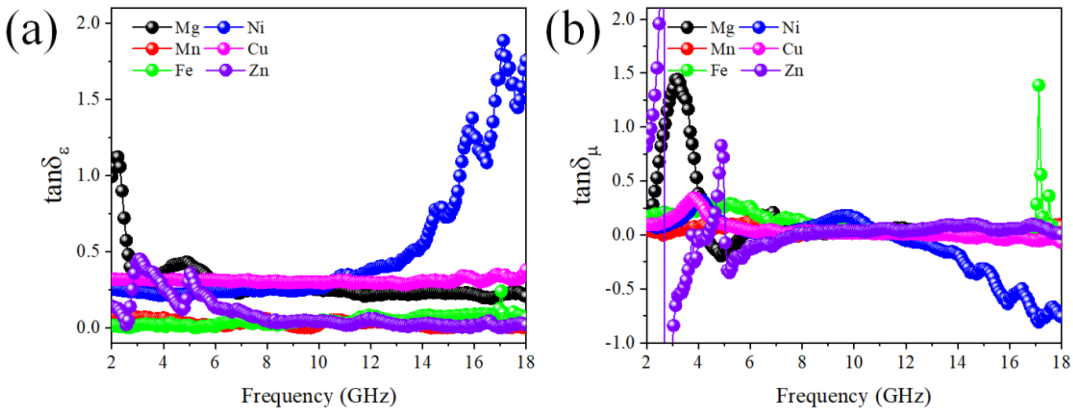

Fig. S11 (a) the dielectric loss tangent and (b) the magnetic loss tangent of the as-obtained samples.

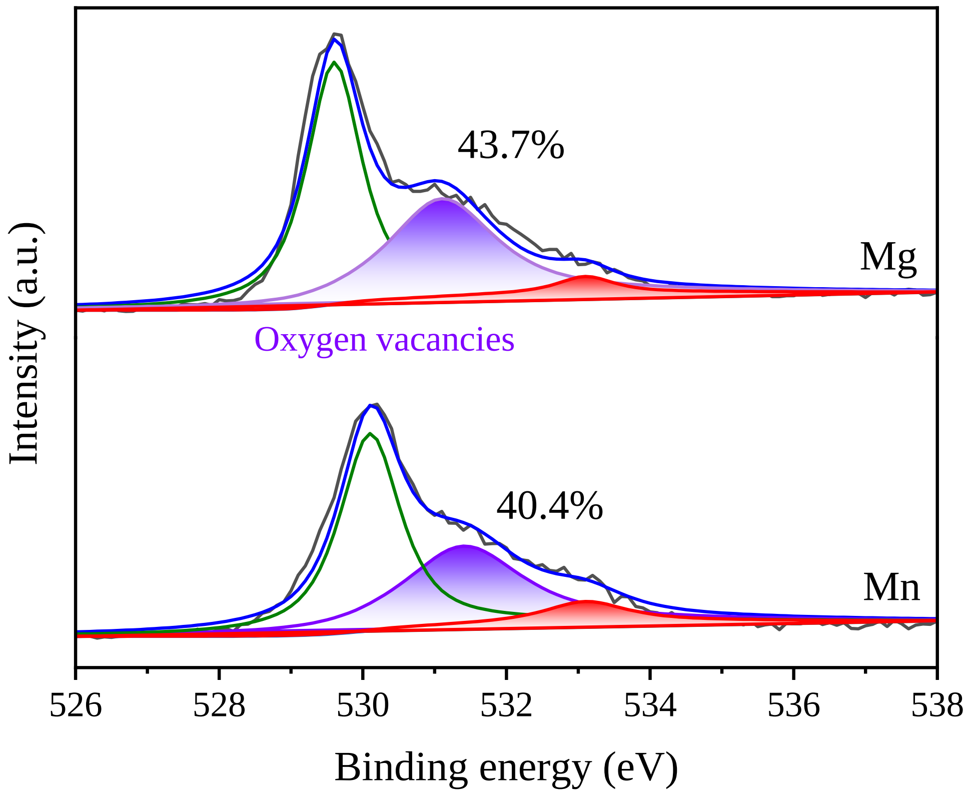

Fig. S12 High-resolution XPS spectrum survey of O 1s of as-prepared samples obtained from divalent metal ions (a) Mg^{2+} and (b) Mn^{2+}.

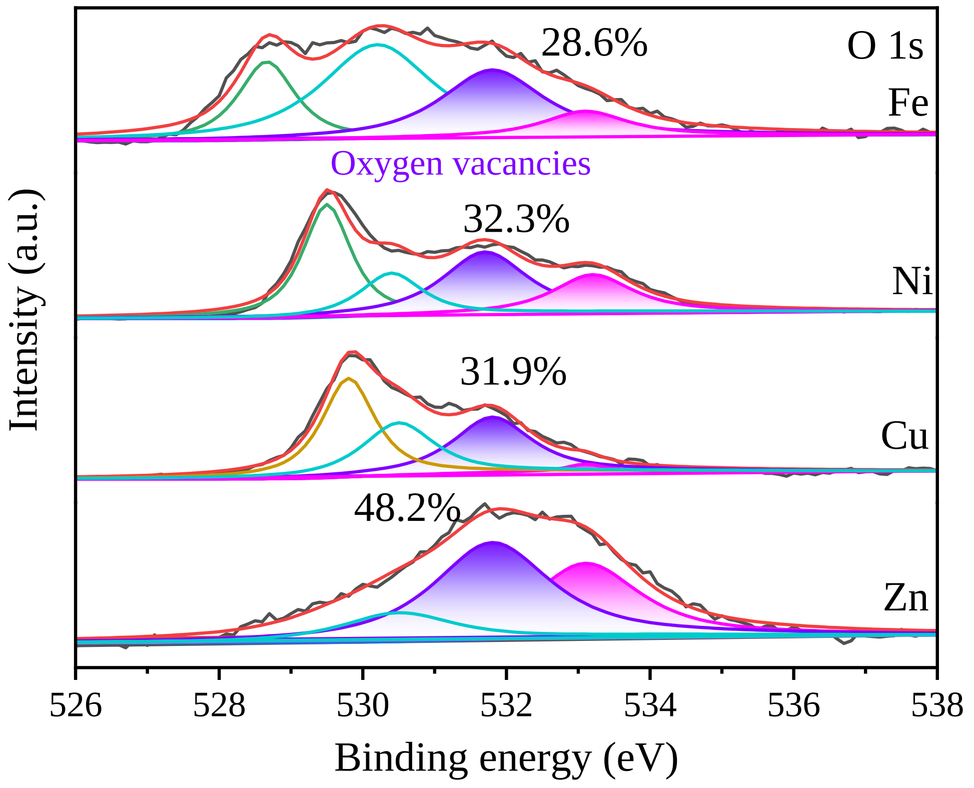

Fig. S13 XPS spectrum survey of O 1s of as-prepared samples obtained from divalent metal ions Fe^{2+}, Ni^{2+}, and Cu^{2+}.

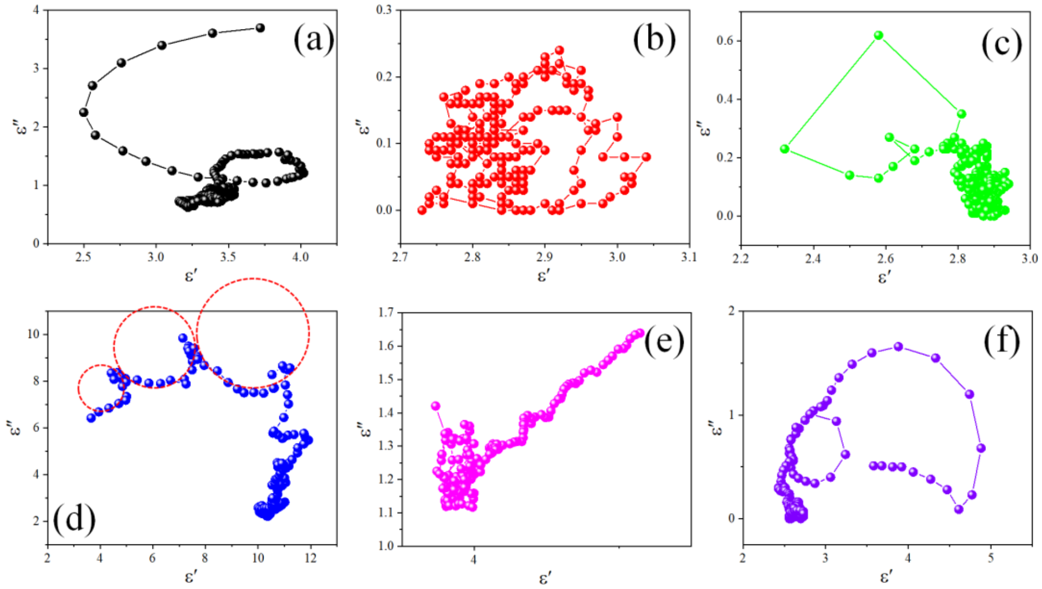

Fig. S14 *Cole-Cole* semicircles of samples obtained from different divalent metal ions

(a) Mg^{2+},(b) Mn^{2+}, (c) Fe^{2+}, (d) Ni^{2+}, (e) Cu^{2+} and (f) Zn^{2+}.
